# Familial Circadian Rhythm Disorder in the Diurnal Primate, *Macaca mulatta*

**DOI:** 10.1371/journal.pone.0033327

**Published:** 2012-03-08

**Authors:** Irina V. Zhdanova, Ken Masuda, Sergey V. Bozhokin, Douglas L. Rosene, Janis González-Martínez, Steven Schettler, Eric Samorodnitsky

**Affiliations:** 1 Department of Anatomy and Neurobiology, Boston University School of Medicine, Boston, Massachusetts, United States of America; 2 Department of Theoretical Physics, St. Petersburg State Polytechnic University, St. Petersburg, Russia; 3 Caribbean Primate Research Center, University of Puerto Rico, San Juan, Puerto Rico; Pennsylvania State University, United States of America

## Abstract

In view of the inverse temporal relationship of central clock activity to physiological or behavioral outputs in diurnal and nocturnal species, understanding the mechanisms and physiological consequences of circadian disorders in humans would benefit from studies in a diurnal animal model, phylogenetically close to humans. Here we report the discovery of the first intrinsic circadian disorder in a family of diurnal non-human primates, the rhesus monkey. The disorder is characterized by a combination of delayed sleep phase, relative to light-dark cycle, mutual desynchrony of intrinsic rhythms of activity, food intake and cognitive performance, enhanced nighttime feeding or, in the extreme case, intrinsic asynchrony. The phenotype is associated with normal length of intrinsic circadian period and requires an intact central clock, as demonstrated by an SCN lesion. Entrainment to different photoperiods or melatonin administration does not eliminate internal desynchrony, though melatonin can temporarily reinstate intrinsic activity rhythms in the animal with intrinsic asynchrony. Entrainment to restricted feeding is highly effective in animals with intrinsic or SCN lesion-induced asynchrony. The large isolated family of rhesus macaques harboring the disorder provides a powerful new tool for translational research of regulatory circuits underlying circadian disorders and their effective treatment.

## Introduction

Our daily schedules are defined mainly by social demands but our body functions rely on the internal circadian clock mechanisms to provide for adaptive synergy of intracellular, physiological and behavioral processes. Mounting evidence indicates that alterations in the circadian clock, leading to misalignment of body rhythms relative to each other and to the environment, is a risk factor for cancer, and for neurological, metabolic and mental disorders [1], [2]. In addition to circadian abnormalities induced by shift work or jet lag, mutations in different genes can lead to sporadic or familial human circadian disorders, reflecting the complexity of the clock and clock-controlled processes, and their diverse targets [3]. Some of human circadian disorders are characterized by phase advance, but many are associated with delay of circadian phase of body rhythms relative to each other or to the light-dark cycle. The delayed sleep phase disorder (DSPD) is the most prevalent known circadian disorder, which is often initiated during adolescence. It manifests as a major stable and involuntary delay of the sleep period relative to the light-dark cycle and socially-desirable activity period [4]. The DSPD is a risk factor for somatic and mental illnesses [5], [6]. Another disorder, the phase delay of food intake rhythm relative to sleep-wake cycle, known as the night eating syndrome (NES), dramatically alters metabolic and endocrine functions [7], [8]. The phase delay in melatonin production relative to sleep time correlates with the severity of major depression [9]. A link between neurological or cognitive alterations and the clock is suggested by the increased incidence of phase delay in patients with attention deficit disorder (ADHD) and the correlation between a polymorphism of the *CLOCK* gene and ADHD [10], [11]. Nevertheless, the whole spectrum of circadian disorders and the mechanisms through which common human ailments might reflect intrinsic desynchrony remain unknown.

The autonomous clock in cells of peripheral tissues requires synchronization and, in mammals, this unifying signal is provided by the *central* clock, the suprachiasmatic nuclei (SCN) of the hypothalamus [12], [13]. The molecular mechanisms of the SCN clock and their relationship to the environmental 24-h cycle are phylogenetically well-conserved. The SCN neurons are active during the day and the principal circadian hormone, melatonin, is produced only at night, independent of a species' nocturnal or diurnal lifestyle. It remains unknown how this similar central signal is then translated into the opposite physiological and behavioral outputs in diurnal and nocturnal species [14]. Given that humans are diurnal, our understanding of the role of the circadian system in human health and disease would benefit greatly from a detailed examination of circadian physiology in diurnal mammals [15], including those phylogenetically close to humans. The rhesus monkey (*Macaca mulatta*) is arguably the best available diurnal laboratory animal for translational research into the organization of the human clock and intrinsic circadian regulation of homeostatic processes. This is, in part, due to its phylogenetic proximity to humans and consolidated nighttime sleep, with electrophysiological patterns similar to those in humans [16], [17], [18], [19], [20].

Here we report the discovery of the first intrinsic circadian disorder in rhesus monkeys, associated with delayed sleep phase, mutual misalignment of behavioral rhythms of activity, food intake and cognitive performance or, in the extreme case, intrinsic asynchrony. The affected animals belong to the same matriline and are part of the large group-M, self-established in 1973 and maintained as isolated community since 1984. This family will now provide a new and unique model to study the molecular and physiological mechanisms of inherent circadian alterations in diurnal primates, and devise effective strategies for treating intrinsic circadian disorders.

## Results

A comparison of behavioral patterns in control rhesus monkeys and group-M animals revealed major differences, as detailed below. The three affected animals belonged to the same matriline, with the M1 and M3 being half-cousins, both with 8 degrees of separation from M2. The circadian phenotypes were largely similar between M1 & M2, while M3 expressed an extreme circadian abnormality. We thus present the data either for (M1 & M2, together) and M3 separately, or for the entire group-M, if changes were common to all three monkeys versus control. The type of comparison provided is identified in the text and figure captions.

### Phase delay in entrained activity rhythms in group-M macaques

**Figure 1:**
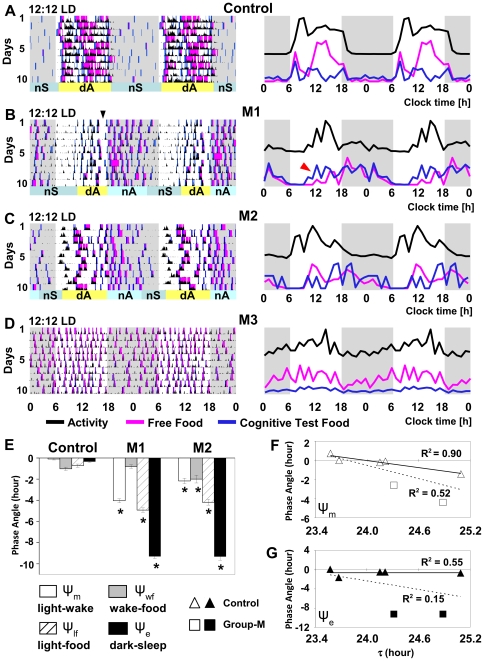
Delayed sleep phase, altered daytime cognitive performance and increased nighttime eating in group-M in LD. **A–D:** Double plots of activity and food intake in 12∶12LD, in (A) representative control (animal C1, τ = 25.1 in CDL) and (B–D) in group-M monkeys, M1, M2 and M3. Left panels – 10-day activity (black), free-food intake (magenta) and food through cognitive test (blue) recordings. Horizontal bars: sleep (blue) and active (yellow) periods; dA – daytime activity, nA – nighttime activity, nS – nighttime sleep. Black arrow head at the top of B-left panel points out a regular end-of-a-day nap (dS); Right panels: corresponding daily profiles, with Y-axis representing change in mean value per hour for each measure shown. Background: white – light period, gray – dark period. Red arrow (B, right panel) - earlier onset of “food through cognitive test” intake, relative to “free-food intake”, reflecting high incidence of incomplete cognitive tests during the day (H). **E** Phase angles in control group and in group-M animals: Ψ_m_, morning phase angle (difference between lights on time and wake); Ψ_wf_, wake-to-feeding phase angle (difference between wake-up time and the first feeding bout of that morning); Ψ_lf_ lights-to-food phase angle (difference between the lights on time and the first feeding bout); Ψ_e_, evening phase angle (difference between the lights off time and consolidated sleep period). *p<0.0001, relative to corresponding measures in control. **F** & **G** Correlation between the intrinsic period (τ) and morning (F) or evening (G) phase angle in control animals (triangle) and M1 & M2 (square). Solid line and R^2^ above it – control only; dashed line and R^2^ below it –control+M1&M2.

In normal rhesus monkeys, we have documented [16] that under entrained conditions of light-dark cycle (LD) and in the absence of other environmental time cues, the daytime activity (dA) is initiated close to the lights-on time, and is associated with high activity levels. Their principal nighttime sleep (nS) episode starts close to the lights-off time and the activity levels remain low throughout the night. Figure 1A illustrates typical patterns of entrained rhythms of activity, food intake and cognitive performance in a representative control animal, with eveningness chronotype. In contrast, in two group-M monkeys (M1 & M2), the onset of dA and nS were significantly delayed (p<0.0001 for both comparisons vs. control) (Fig. 1 B,C), with corresponding increase in the morning and evening phase angles, Ψ_m_ and Ψ_e_, reflecting this abnormality in entrainment to LD cycle (Fig. 1E–G).

The activity levels in M1 & M2 reached peak during dA, as in control (Fig. 1A–C). At the end of the light period, they frequently displayed a relatively short daytime sleep (dS) episode, lasting 1–3 h (Fig. 1 B, black arrow head). The dS typically ended right before the lights-off time and was followed by prolonged nighttime activity (nA) of modest intensity, significantly lower than that during dA (p<0.0001; Fig. 1B,C). The consolidated nS period was initiated close to the end of the dark period and continued into the early lights-on hours, with the overall nS duration being significantly shorter than in control (p<0.001).

In contrast to M1 & M2, the third family member (M3) exhibited an entrained rhythm of activity characterized by very low amplitude (p<0.0001 vs. control or M1/M2) (Fig. 1D). Increased fragmentation of activity rhythm in this animal made it difficult to evaluate his phase angles, though no major phase shift in activity patterns could be observed in M3 under LD conditions.

**Figure 2:**
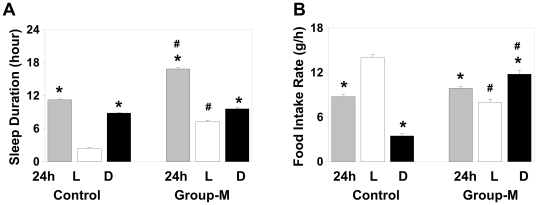
Changes in day-night variation in sleep and food intake in Group-M monkeys. **A** Sleep duration, total (gray), daytime (white) and nighttime (black), in representative Control and Group-M monkey (M1) (as seen in Fig. 1A, B) illustrate that increase in total sleep in Group-M is due to delayed awakening and increased sleep during the day, as per extreme eveningness phenotype. **B** In Group-M animals (shown in M1), the rate of food intake (per hour) is relatively low at daytime (white), when compared to total 24 h (gray) or nighttime (black) rate of their food consumption, or vs. control. In contrast, their nighttime food intake is significantly increased, relative to control. Mean value (SEM) in representative animals; *p<0.01 vs. its own daytime value; # p<0.01 vs. corresponding measure in control.

Relative to control, the overall group-M displayed significant reduction in daily activity amplitude (p<0.01), increase in daytime sleep duration and total sleep time over a 24-h period (p<0.01, for these three comparisons; Fig. 2A).

### Phase delay in feeding initiation and increase in nighttime eating in group-M

Total 24-h food intake in group-M monkeys was not significantly different from that in control group. However, both M1 & M2 demonstrated significantly delayed onset of food intake relative to lights on time (Ψ*_lf_*, *p*<0.0001), consistent with the delay in Ψ*_m_*, and M2 displayed a delay in initiating food intake relative to wake-up time, Ψ*_wf_* (Fig. 1E–G). The daytime food consumption in M1 & M2 constituted only 39.4±2.81% of total 24-h food intake (vs. 90.1±2.47% in control, p<0.0001). In contrast, they were eating throughout most of the night, until right before falling asleep in the early morning hours, with the overall nighttime food consumption significantly higher than at daytime (p<0.0001), or relative to nighttime food intake in control (p<0.0001, Fig. 2B). As a result, peak locomotor activity levels during the day corresponded to lower food intake, while relatively low nighttime activity levels were associated with increased food intake in M1 & M2; an inverse pattern to that in control (Fig. 1A–C). Similarly, in M3, the daytime food consumption was significantly reduced (53.8±1.79% of total food intake) and the nighttime food intake was increased (p<0.0001 vs. control for both comparisons). Based on the wavelet analysis, group-M monkeys had an overall low correlation between activity and food intake rhythms: r = 0.41 in M3, 0.63 in M1 & M2 vs. 0.92 in control).

### Altered patterns of cognitive performance in group-M

The control animals had no significant difference between daytime and nighttime cognitive performance measures in delayed match to sample test (DMST), although the number of cognitive tests was significantly (p<0.001) lower at night than during the day, consistent with their overall lower nighttime food intake (Fig. 1H). In contrast, during the day, all three group-M animals displayed lower success rate and lower percentage of completion of initiated tests (p<0.01 vs. night, for both comparisons), and this was especially robust in M1 & M2. Since incomplete DMST prevented access to “free food” (see Methods), animals continued initiating cognitive tests and, typically, more food during the day was consumed through DMST than through the “free food” paradigm (p<0.05). Thereafter, at night, cognitive performance in group-M was improved (p<0.05 vs. daytime) and the percent tests completed was increased, followed by augmented “free food” intake (Fig. 1B). Improved nighttime performance led to the overall 24-h cognitive performance measures in group-M being superior to those in control, as per higher overall success rate and percent tests completed, and shorter reaction times (p<0.05 for all three comparisons). Together, this indicated that although the daily patterns of cognitive performance were impaired in group-M, the cognitive function *per se* remained intact.

### Circadian rhythm abnormality in group-M monkeys is intrinsic

**Figure 3:**
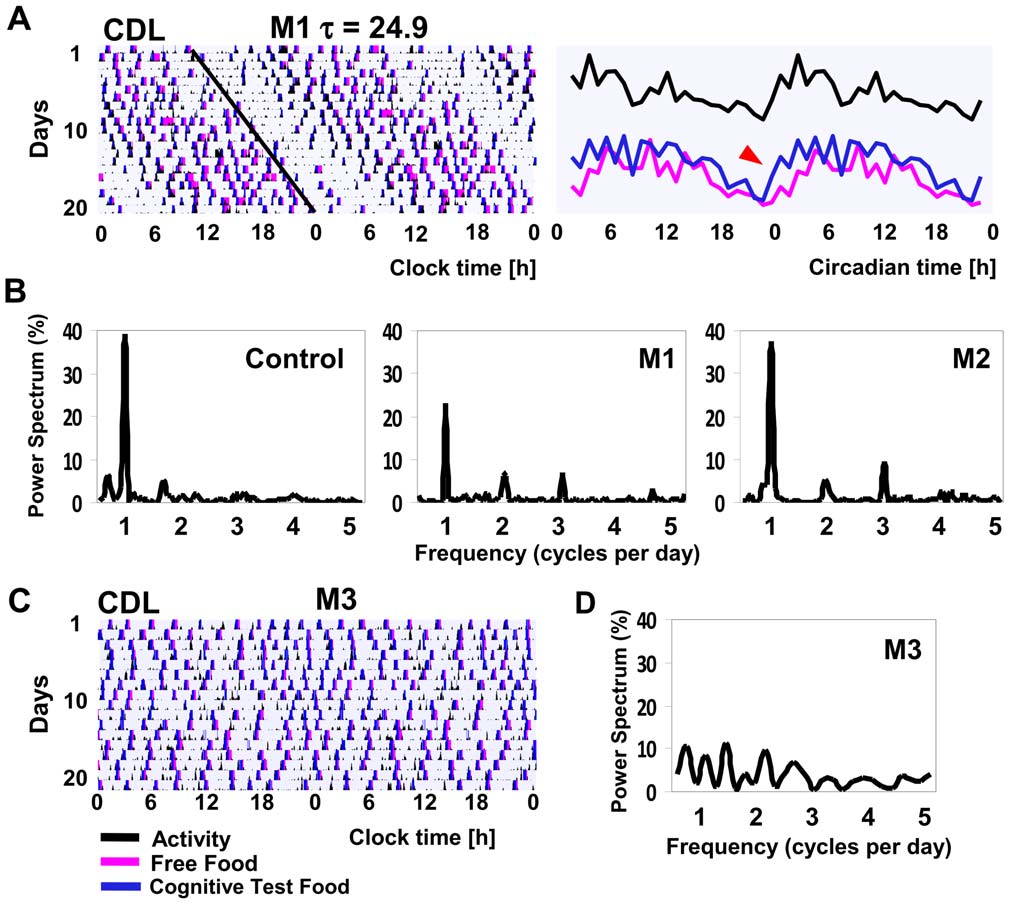
The intrinsic rhythms of activity and food intake in group-M under constant dim light conditions. **A** Left: Stable intrinsic rhythm of activity in monkey M1, with intrinsic circadian period τ = 24.9 h (22 days, shown per Clock Time). Right: corresponding mean patterns of food intake and cognitive performance (mean data profiles, shown per hour of Circadian Time, with CT0 = onset of activity). Red arrow (right panel) - earlier onset of “food through cognitive test” intake, relative to “free-food intake”, reflecting high incidence of incomplete cognitive tests during the day. **B,D** Power spectrum of activity rhythms within the 0.5–5 cycles per day frequencies (100% = the sum of all the powers shown) for control, M1, M2 and M3. **C** Raw data recording over 22 days in monkey M3 (shown per Clock Time), illustrating intrinsic asynchrony. Color scheme as in Fig. 1.

Removal of all environmental time cues under constant dim light (CDL) conditions revealed that the phenotypes observed in LD are intrinsic in nature. In M1 & M2 animals that displayed a stable intrinsic rhythm in CDL (Fig. 3A, left), increased activity level at the beginning of subjective day remained associated with altered cognitive performance and food intake, as during the dA component in LD (Fig. 3A, right). This was followed by reduced activity and improved cognitive performance, as in nA in LD (Fig. 1B). Notably, a nap typically separating dA and nA components in LD (dS) disappeared in CDL, consolidating these two distinct components into a continuous active period, and suggesting that the unusual split in the activity period in these group-M animals was light-dependent.

Sleep and food intake in CDL were modestly increased (p<0.05, for both comparisons vs. LD). When the entire frequency range of intrinsic oscillations was evaluated using Fourier transform, group-M animals showed significantly reduced power of circadian and increased power of high frequency oscillations (p<0.0001 for both, vs. control). However, within a smaller range of frequencies, from 0.5 to 5 cycles per day, the relative power of the circadian oscillation in M1 & M2 was comparable to control (Fig. 3B). Remarkably, unlike any rhesus monkey studied in our laboratory, young or aged, animal M3 manifested complete circadian asynchrony in CDL (Fig. 3C,D).

### Circadian disorder in group-M monkeys is associated with normal length of intrinsic period

In control rhesus monkeys, the intrinsic period (τ) ranged from 23.5 h to 25.1 h, as per our earlier report [16]. In group-M monkeys, τ remained within the control range (24.3–24.9 h). This indicated that the circadian disorder in group-M is not a result of an unusually short or long intrinsic period. Similar to that found in humans [21], in control, τ positively correlated with increase in Ψ_m_ (p<0.01), which was most pronounced in the animal C1, with τ = 25.1 h (shown in Fig. 1A). When M1 & M2 were added to the analysis, the significant correlation between τ and Ψ_m_ was lost (p = 0.06; Fig. 1F) and no correlation between τ and Ψ_e_ was documented (p = 0.33, Fig. 1G).

### Melatonin can entrain rhythms in group-M macaques, but cannot eliminate circadian rhythm dissociation

**Figure 4:**
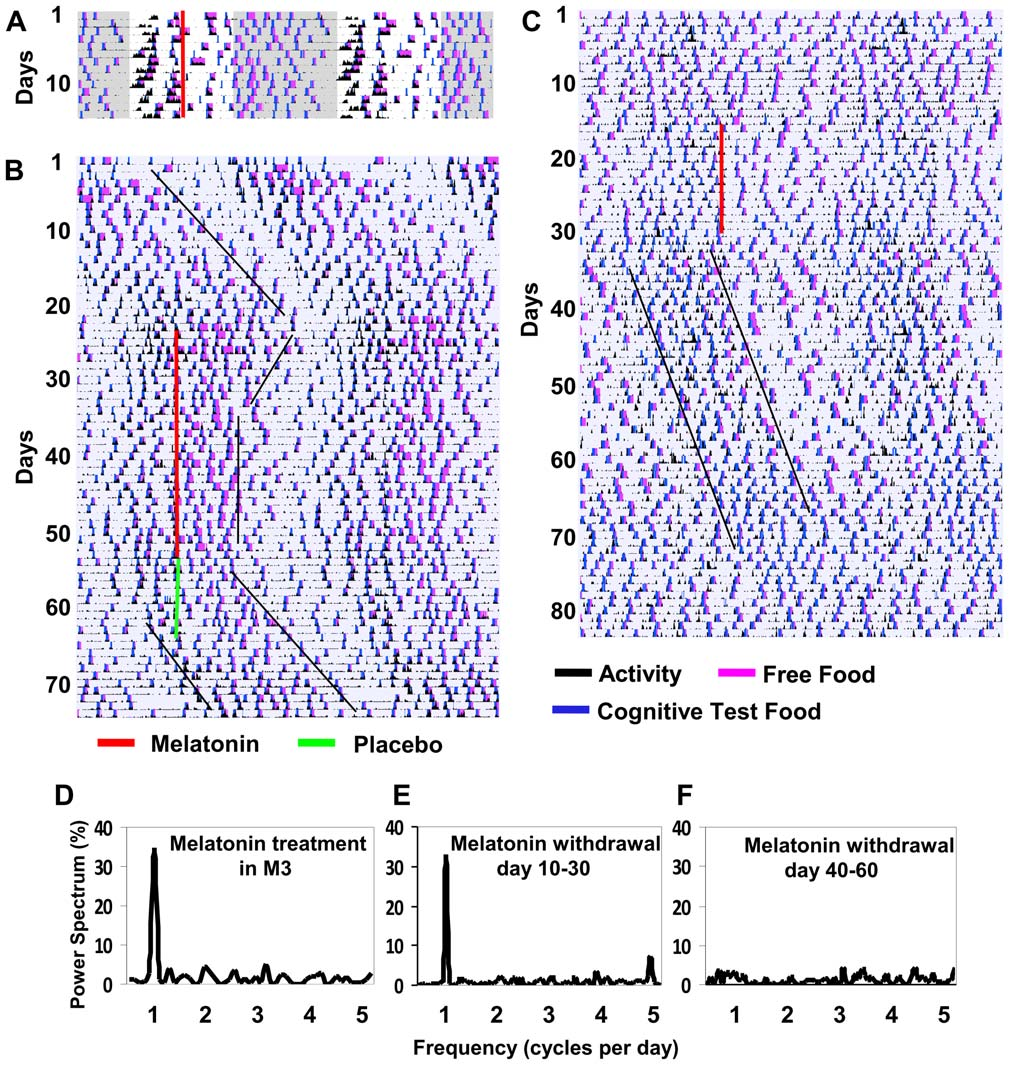
Effects of melatonin in group-M: phase shift, entrainment, sleep-promotion and reinstatement of intrinsic rhythm.

There are no established treatments for human circadian disorders [4]. It has been suggested, however, that melatonin, the pineal hormone playing a major role in the circadian system, can be helpful due to its ability to shift and entrain the phase of circadian rhythms in humans [22], [23]. We have reported earlier that melatonin can acutely promote sleep in rhesus monkeys [24], [25], as in other diurnal species [26], and can shift and entrain their circadian rhythms in a phase- and τ-dependent manner [16]. Similarly, in group-M animals, daytime melatonin administration in LD could shift the phase of daily rhythms (Fig. 4A) and increase the daytime and 24-h sleep time (p<0.01, for both comparisons).

When given in CDL, melatonin entrained the intrinsic rhythms to a 24-h period in M1 and M2 (Fig. 4B). The phase of entrainment to melatonin coincided with the beginning of the animals' subjective night, as typical of control monkeys with τ over 24 h [16]. However, in M1 & M2, entrainment by melatonin did not modify the unique phase relationship between the activity, food intake and cognitive performance patterns, which retained mutual misalignment, with increased food intake during subjective night (p<0.001). Withdrawal of melatonin, followed by placebo or no treatment in CDL, led to reinstatement of the intrinsic period in both control [16] and M1 & M2 monkeys (Fig. 4B).

### Melatonin can temporarily reinstate intrinsic activity rhythm in intrinsically asynchronous animal

The monkey with original intrinsic asynchrony (M3) also entrained to melatonin in CDL (Fig. 4C). Remarkably, following melatonin withdrawal, M3 exhibited an intrinsic circadian rhythm of activity and food intake (τ = 24.3 h; Fig. 4D), albeit of low amplitude, and maintained this rhythm for over 30 days in CDL (Fig. 4E). Thereafter, a gradual decline in the power of his circadian rhythm reverted to circadian asynchrony (Fig. 4F). This established that M3 has the capacity to generate an intrinsic circadian rhythm and suggested that repeated melatonin treatment can have a long lasting consolidating effect on the intrinsic circadian mechanisms in this animal.

### Different photoperiods can entrain group-M macaques but do not eliminate misalignment of their daily rhythms

**Figure 5:**
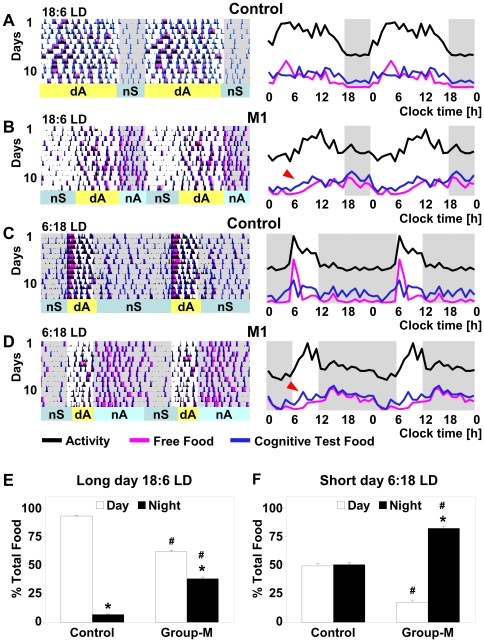
Persistence of characteristic circadian group-M patterns of activity and food intake in long and short day environment. **A** & **B** Long day, 18∶6 LD, in control (**A**; animal C1, as in Fig. 1A) and M1 (**B**). Color scheme as in Fig. 1. **C** & **D** Short day, 6∶18 LD, in control (**C**, animal C1) and M1 (**D**). **E** & **F** Change in daytime (white) and nighttime (black) food intake (% of total food intake per 24 h) in representative animals (shown in A–D). Mean value (SEM); *p<0.01 vs. its own daytime value; # p<0.01 vs. corresponding measure in control.

We then evaluated whether the unusual daily patterns in group-M animals could be modified by different photoperiods, and exposed them to long day (18∶6 LD) and short day (6∶18 LD) conditions (Fig. 5). The speed of entrainment to these different LD schedules did not differ in control and M1 & M2 monkeys (data not shown), and the duration of the subjective day and subjective night behavior significantly correlated with the length of the light and dark periods, respectively (R^2^ = 0.86 and 0.85 in M1 & M2). However, independent of the illumination schedule, the M1 & M2 monkeys retained their characteristic phase delay relative to light onset and their altered relationship between activity, food intake and DMST performance (Fig. 5B,D). This resulted in increased Ψ_m_ and Ψ_e_, and a shorter dA period, when compared to control tested in parallel (p<0.0001; for all three comparisons). The total sleep time and total food intake remained similar under three different LD cycles (data not shown). However, characteristic of group-M day-night difference in food consumption modified their behavior under both long and short day conditions in favor of nighttime feeding, as illustrated in Figure 5E,F. As in 12∶12LD, cognitive performance in M1 & M2 was improved at night (p<0.05 vs. daytime). Consistent with a longer night duration in 6∶18 LD, this led to the highest 24-h performance measures in group-M during this short-day and long-night schedule (p<0.01 vs. other LD cycles). Such effect was not present in control group. In the asynchronous monkey M3, weak entrainment was achieved under the short, but not long day schedule. Unlike that following entrainment to melatonin, entrainment to different photoperiods failed to reinstate the intrinsic circadian rhythmicity in M3.

### Restricted feeding (RF) is a powerful entraining factor in group-M macaques

**Figure 6:**
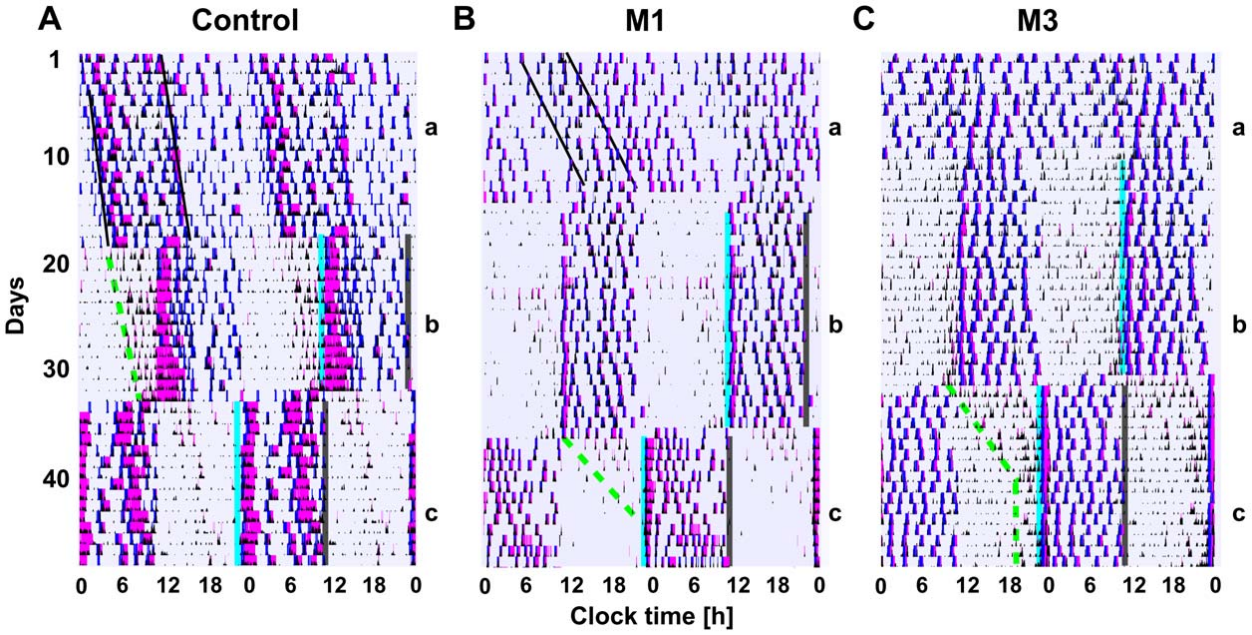
Entrainment to restricted feeding (RF) in control and group-M monkeys. **A–C:** Continuous recordings in CDL, conducted in parallel over a 50-day period, in a control animal C6 (A), M1 (B) and M3 (C), illustrating free-running during around-the-clock food access in control and M1, and asynchrony in M3 (a), effective entrainment to RF (b), and re-entrainment to a new RF schedule (c). Blue line - onset of food access, black line - end of food access. Dashed green line – depicts an approximate dynamic of anticipatory activity during transition between food access schedules. Color scheme as in Fig. 1.

Similar to that in the control animals, RF limited to 12 consecutive hours per day led to expedient change in activity, sleep and food intake patterns in all three group-M animals, entraining them to 24-h period (Fig. 6), reducing their total food consumption, and increasing sleep time (p<0.01 for both comparisons vs. unrestricted feeding). Animals displayed the anticipatory activity closer to food availability time, as per documented increase in unrewarded touches of the computer screen 1 h before food availability (vs. 2 or 3 h prior; p<0.05, for both comparisons). In rodents, presence of endogenous food entrainable oscillator (FEO) is typically documented while exposing animals to several days of fasting, following RF, so as to reveal a free-running rhythm of food anticipatory activity (FAA) and exclude a masking effect of a homeostatic “hour glass” mechanism. This methodological approach cannot be used in humans or non-human primates. However, a gradual shift in anticipatory behavior following abrupt phase delay in RF schedule suggested “true” entrainment, especially notable in intrinsically asynchronous M3 (Fig. 6C).

### Intact SCN is required for the phenotype observed in group-M monkeys

**Figure 7:**
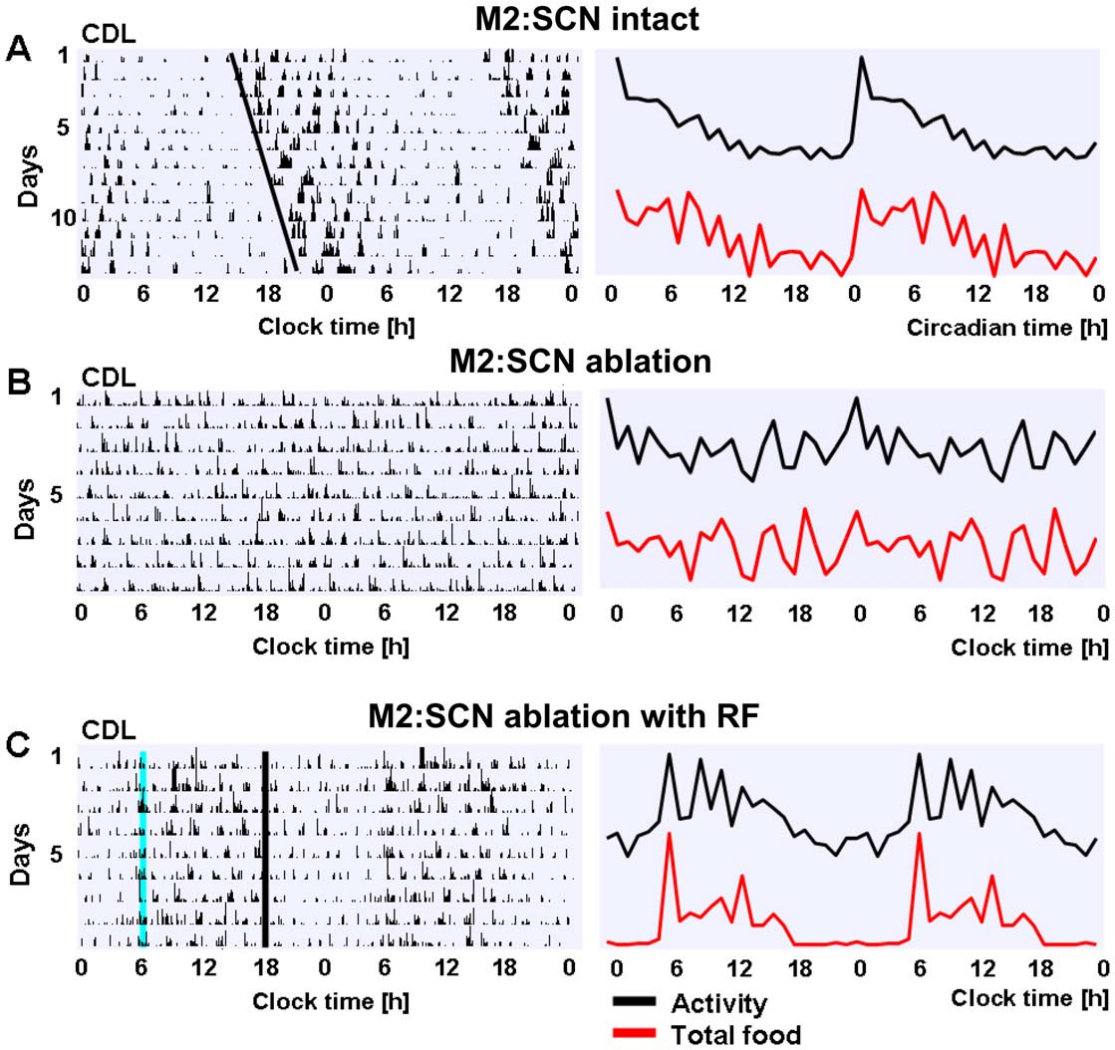
SCN ablation eliminates intrinsic circadian patterns of activity and food intake in group-M. **A** & **B** Double plots of continuous activity recording (left) and corresponding daily profiles (mean value per hour; right panel) of activity (black) and total food intake (red) in M2 before (A) and after (B) electrolytic ablation of the SCN. Black line in A (left plot) highlights the intrinsic free-running rhythm in CDL (τ = 24.3 h), with no such rhythm following the SCN ablation (B). **C** Similar plots during the period of restricted feeding (RF) in CDL, illustrating entrainment to 24-h period of food availability following SCN lesion. Blue line - onset of food access, black line - end of food access.

To determine the extent to which the central clock, located in the SCN, is required for the phenotype observed, we conducted a bilateral electrolytic SCN ablation (SCNx) in M2 (Fig. 7), an animal with phase delay in LD (Fig. 1C) and stable intrinsic rhythm in CDL (Fig. 7A). This led to complete loss of circadian rhythms of activity and food intake (Fig. 7B).

**Figure 8:**
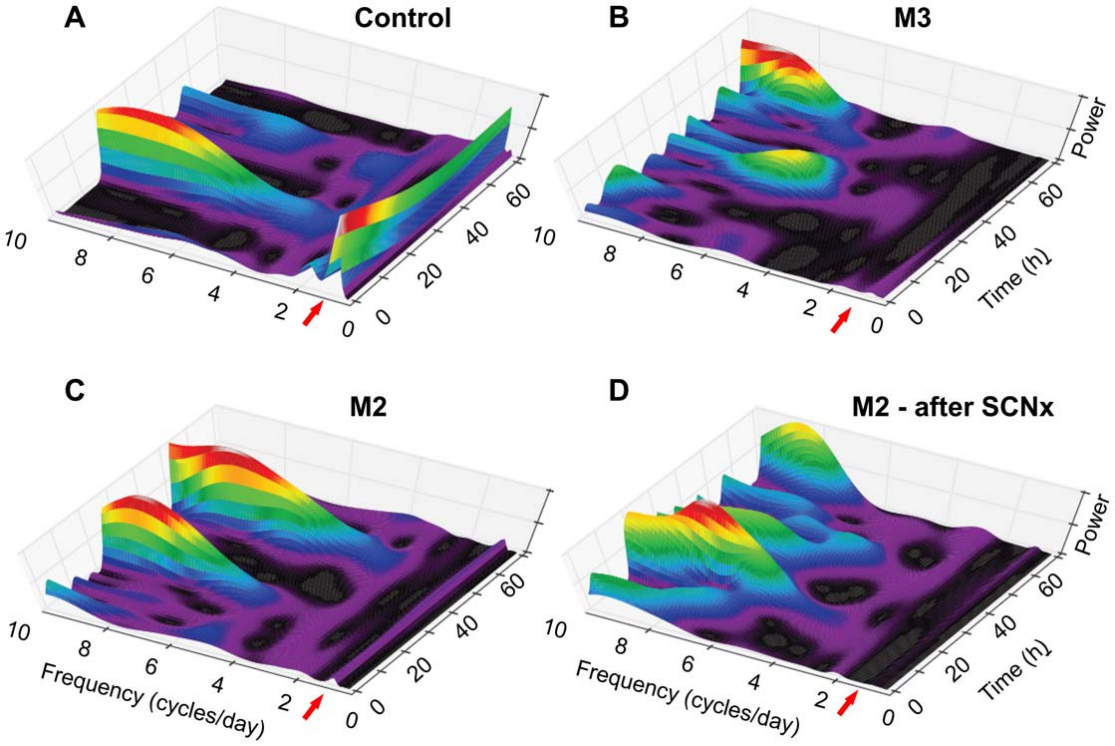
Similarities between dynamic patterns of spontaneous circadian asynchrony and that following the SCNx in group-M. The dynamic changes in the power of intrinsic circadian and ultradian rhythms of activity in CDL are reflected in 3D time-by-frequency wavelet maps of a 70-h period of recording for a representative control (**A**), a group-M animal (M3) with intrinsic circadian asynchrony (**B**), and a group-M animal (M2) with initially preserved circadian rhythm (**C**), which was lost following SCN ablation (**D**). Red arrows point to circadian frequency (∼1 cycle per day). It is continuously present in control and intact M2 (A & C), with ultradian rhythms, especially those with a frequency of more than 6 cycles per day, being prevalent during the active period of subjective day (wavelet ridges: blue-red), and typically low or absent during subjective night (wavelet valleys: purple-black). In contrast, in animals with circadian asynchrony due to familial circadian disorder (B) or SCN ablation (D), the lack of circadian frequency is associated with frequent high-frequency wavelet ridges, which do not follow a circadian pattern. X axis – frequency (cycles per day), Y axis – time (h), Z axis – relative spectral power, reflecting the power of all detected frequencies being 100%, i.e., including those beyond what is shown on the X-axis.

A detailed analysis, using the time-frequency wavelet transform (Fig. 8) revealed that following SCNx the behavioral patterns in M2 became remarkably similar to those in intact M3 (Fig. 8B,D). Both animals lacked stable circadian rhythm and periodic (daily) occurrence of ultradian frequency ridges characteristic of control subjects in CDL (Fig. 8A) and of M2 prior to SCNx (Fig. 8C). Similar to M3, M2 preserved its ability to entrain to RF (Fig. 7C), consistent with prior evidence of the FEO being independent of the SCN [27].

## Discussion

This report describes the behavioral phenotype of the first intrinsic circadian disorder documented in diurnal non-human primates. It is characterized by either delayed phase, with major misalignment of entrained and intrinsic rhythms of activity, food intake and cognitive performance or, in the extreme case, by intrinsic asynchrony. The three affected animals belong to the same matriline, with M1 and M3 being half-cousins, based on the common maternal grand-father, in addition to their 2 degrees of separation along the matriline. There are 8 degrees of matrilineal separation between M1/M3 and M2, and no known patrilineal relationship. This suggests that the identified disorder is familial in nature, considering that no other young or aged rhesus monkey studied in our laboratory over the years had any of the phenotypic characteristics of group-M. This is in spite of all these control animals being originally exposed to similar environmental conditions and daily feeding schedules of primate facilities, whether being raised in the same CPRC colony or other NIH-funded primate centers. We, however, refrain from speculating at this point as to which genetic abnormalities could potentially lead to the observed phase delay and other behavioral changes in group-M, especially in view of inconsistent results on the genetic bases of delayed sleep phase in humans [28], [29], [30], [31], [32]. Our further investigation of the circadian phenotype and genotype in these and other group-M family members (n = 256) in the newly-constructed high throughput primate circadian laboratory should clarify the inheritance pattern and molecular abnormality underlying the disease.

The circadian phenotype of group-M monkeys shares common features with two human disorders, DSPD and NES [4], [5], [33]. Although these disorders have distinct non-overlapping manifestations, the extent to which they might share some phenotypic features and intrinsic mechanisms remains unknown. Similar to DSPD, group-M animals demonstrate delayed circadian phase of entrainment to light. Although this is reflected in the late onset of both daily rhythms of activity and food intake, there is a distinct temporal dissociation between these two rhythms. Unlike in control, the daytime peak in activity levels is associated with low food intake in group-M monkeys. In contrast, decline in activity levels at night, under both entrained and constant conditions, is associated with increased food intake in group-M, similar to that in humans suffering from NES. Moreover, the daily rhythm of cognitive performance in these group-M monkeys is also affected, being compromised at daytime and remarkably improved at night. It remains to be seen whether this is due to alterations in signal perception, sensorimotor control, memory function, attention span or motivation. To our knowledge, cognitive performance patterns in DSPD and NES patients have not yet been studied, and feeding patterns in DSPD patients have not been systematically addressed. However, increased incidence of phase delay in ADHD patients and a reported association of this disorder with a polymorphism in one of the core clock genes [10], [11], warrants further in depth investigation of a link between cognitive and circadian functions.

In view of our findings that restricted timing of food availability has robust entraining effect in all the animals, with phase delay (M1 & M2), intrinsic (M3) or induced (SCNx M2) asynchrony, the RF could represent an effective treatment strategy for a number of circadian disorders in humans, including DSPD and NES. Interestingly, the two most recommended treatment strategies for circadian disorders, light schedule and melatonin administration, proved to be less effective in the group-M circadian disorder. Both treatments were highly efficient in entraining the activity and feeding rhythms to 24-h period. However, mutual alignment of these internal rhythms remained altered, daytime cognitive performance continued to be inferior to that at night, and increased nighttime eating persisted. Even extending a photophase to 18 h, leaving only 6 hours of darkness, did not modify the nighttime eating preference and, instead, moved the entire consolidated sleep episode (nS) to morning hours (Fig. 5B). It remains to be seen whether light of different intensity or spectral composition could be more effective in synchronizing the internal rhythms affected by the intrinsic circadian disorder. Similarly, more research on the effects of melatonin in different types of intrinsic circadian desynchrony is needed. Our finding that repeated melatonin treatment was able to reinstate intrinsic rhythm of activity in the animal with severe circadian asynchrony is very promising (Fig. 4C).

Circadian disorders can stem from diverse alterations in upstream, downstream or within-the-clock mechanisms. Normal visual acuity and lack of free-running rhythm under entrained conditions ruled out a deficiency in the upstream visual input to the central clock in group-M. We could also exclude that an altered phase angle of entrainment to light resulted from unusual intrinsic period length, as often observed in mice with mutations in core clock genes [34]. Studying circadian rhythms in humans is difficult and little is known about intrinsic periods in patients with circadian disorders. For example, a study that characterized the first familial advanced sleep phase disorder (ASPD) was able to evaluate the intrinsic period in only one of the affected family members and found it to be short [35]. A study in one DSPD patient suggested an abnormally long intrinsic period [36]. No data on the intrinsic periods in NES patients have been reported, so far. The group-M phenotype suggests, however, that at least some circadian disorders in diurnal primates, including those with delayed phase and nighttime eating, can be unrelated to the abnormal length of the intrinsic period.

A coexistence of the master clock, SCN, and active circadian oscillators independent of the SCN and not being directly regulated by the environmental illumination, inevitably poses a question of whether certain circadian disorder can result from alterations at either of these levels or both. Mutations in core clock genes are most likely to affect both central and peripheral oscillators, however mutations in tissue-specific clock-controlled genes or transcription factors might have a more variable impact. As a first step in addressing this issue for the circadian disorder in group-M, we determined whether circadian oscillators, left without the integrating control of the SCN, would be able to sustain the behavioral phenotype observed. Loss of circadian synchrony after SCN ablation in M2, an animal with delayed phase of activity and food intake, but robust circadian period prior to surgery, indicated that SCN integrity is required and preserved activity of peripheral oscillators is not sufficient for the phenotype presented by monkeys M1 and M2. Subjecting the SCNx M2 to RF not only entrained its rhythms but also eliminated the desynchrony between activity and food intake rhythms. This allows us to suggest that the intrinsic desynchrony observed in M1 & M2 is SCN dependent and may result from either miscommunication within the SCN or between the SCN and peripheral oscillators, not entrained by light. The third animal of group-M (M3) demonstrated lack of intrinsic circadian rhythmicity and attenuated entrainment to light. This could be due to lack of active SCN, as in M2 following SCN ablation. However, a preserved entrainment to melatonin in M3 and temporal emergence of intrinsic circadian rhythm after melatonin withdrawal, with neither effect observed following SCN lesion in M2, suggest that SCN in M3 is preserved but its activity is significantly impaired.

Based on this initial investigation of the circadian disorder in group-M macaques, we hypothesize that it might stem from inherent reduction in the amplitude of circadian oscillations, altering the coupling of individual pacemakers within their central clock, a phenomenon demonstrated in *in vitro* studies in rodents [37], [38]. Uncoupling of distinct SCN subregions in normal rodents [39], induced by prolonged light exposure (which inhibits the circadian system), can manifest as a split of the activity rhythm into two oscillations with similar or different frequency and variable phase angle, referred to as “morning and evening oscillators” [40], [41]. An intrinsic condition with increased phase angle between activation of distinct areas of the SCN, or between the SCN and downstream targets, including peripheral oscillators, might underlie a split of the activity period into daytime (dA) and nighttime (nA) components, often separated by nap (dS). Similarly, this could explain altered phase angle of entrainment to light, and mutual misalignment of activity, food intake and cognitive performance patterns in M1 & M2 animals. Moreover, weak circadian input could lead to secondary, feedback processes originating in physiological targets of circadian regulation, further promoting desynchrony [42].

A more pronounced alteration in internal coupling could then underlie intrinsic asynchrony in M3. Notably, mutual synchrony in SCN cell preparations could be restored after prolonged inhibition of their activity [43]. Melatonin is one of the established inhibitors of SCN neuronal activity [44], [45]. Hence, the melatonin effect in M3 could be potentially due to temporal restoration of internal coupling following repeated inhibition of the SCN. Loss of circadian rhythmicity in rodents homozygous for circadian gene mutations, with preserved intrinsic periodicity in heterozygous animals (for review, [34]), might also suggest that M3 phenotype could reflect homozygocity. This issue will be addressed through phenotypic and genotypic evaluations of M1 and M3, their recently obtained progeny, and other family members of group-M.

Further behavioral, molecular and neurophysiological studies will address the nature of the discovered circadian disorder in group-M family. Considering that dynamic intrinsic processes are impossible to study in the human SCN, the rhesus monkey could provide a uniquely appropriate model to address the phenomenon of circadian uncoupling in diurnal primates. Moreover, studying this, so far one-of-a-kind rhesus monkey family harboring a circadian disorder, has outstanding potential for providing insight into the ways circadian oscillators, both central and peripheral, interact, entrain and define the daily propensity for homeostatic processes in diurnal primates, including humans.

## Materials and Methods

### Ethics Statement

The studies were performed in accordance with the recommendations of the Weatherall report “The use of non-human primates in research”. All procedures were approved by the Institutional Animal Care and Use Committee of Boston University Medical Campus (AN-14560), and conformed to the NIH *Guide for the Care and Use of Laboratory Animals* and the U.S. *Public Health Service Policy on Humane Care and Use of Laboratory Animals*.

### Subjects

Eight young adult (5.6–6.5 years of age) male rhesus monkeys (*Macaca mulatta* of Indian origin) were used. Four of the animals originated from the Caribbean Primate Research Center, including three related animals belonging to group-M and one unrelated monkey that served as within-colony control (C6). The rest of the animals, studied in parallel with group-M, were obtained from different primate center colonies within the US. These animals were not previously exposed to any experimental manipulations, including sleep or circadian studies. The animals had normal daily (i.e., documented in light-dark cycle) and circadian (i.e., documented in constant dim light conditions) activity and food intake patterns, as described earlier (Masuda & Zhdanova, 2010). The subjects not associated with group-M are referred to as controls in the text.

All eight subjects were in good health, as determined by regular veterinary screening. Lack of occult pathology in the brain was confirmed via Magnetic Resonance Imaging (MRI) scans (Intera 3T Scanner, Phillips Instruments, North Andover, MA, USA). The monkeys were continuously monitored via two video cameras per individual housing chamber and were briefly inspected by the research staff on a daily basis, at random hours, to avoid entrainment. No new unusual behaviors (e.g., stereotypy, aggression, altered food intake, etc.) were documented throughout the period of investigation, suggesting a lack of negative impact of temporal isolation on adult rhesus monkeys, as per earlier reports [16], [20].

### Housing and enrichment

The experimental sleep/circadian laboratory is maintained within the Laboratory Animal Science Center at Boston University Medical Center (BUMC), fully accredited by the Association for the Assessment and Accreditation of Laboratory Animal Care (AAALAC).

In order to unmask individual behavioral patterns, animals were housed in individual oversized custom-designed stainless steel primate cages (100×100×130 cm), each within an individual light-controlled and sound-attenuated circadian chamber for uncovering intrinsic circadian rhythms. Each of six chambers was equipped with air conditioning units, temperature and humidity sensors. Temperature was maintained at 22–24°C and mean humidity levels were 36%.

The goal-oriented behavior, described in the Feeding and Cognitive test sections, and associated with positive reinforcement proved to be a powerful enrichment technique. In addition, diverse rubber/plastic toys and a “primate mirror” were placed in the chamber.

### Environmental illumination

Depending on the experimental period, animals were maintained under a 12∶12 h, 18∶6 h or 6∶18 h light∶dark cycle (LD, ∼100 lux: 0.1 lux) or constant dim light (CDL, <10 lux). Incomplete darkness in LD (<0.1 lux) was due to the presence of a dimmed touch-screen computer monitor in each chamber, with dark background and a gray moving target rectangle, allowing animals to self-administer food (as detailed below). The bright lighting was provided by two wall-mounted fluorescent lamps (24″ 20 Watt T12 Natural Spectrum, Philips Lighting). Dim light was delivered by “rope light” (24″, 1″ bulb spacing, 0.2 Watt each, Utilitech). Light was transmitted through the plastic cage ceiling (Lexan, GE Plastics, Pittsfield, MA).

### Feeding

Each circadian chamber was equipped with an individual touch-screen monitor, connected to an automatic pellet dispenser (ENV-203-1000, Med Associates Inc., St. Albans, VT). This allowed for *ad libitum* around the clock self-administration of 1 g food pellets (Nutritionally complete dustless precision pellets, Bio-Serv, Frenchtown, NJ) via pressing a moving on-screen target. This food access paradigm was called “free-food” and the time each pellet dispensed was documented.

The “free-food” paradigm was also used to evaluate the subject's visual acuity, since it can impact circadian entrainment to light-dark cycle and quality of cognitive performance. To achieve this, the gray-color moving target was gradually reduced in size until the percent of incorrect touches of the screen reached significance. This threshold target size was compared between the control and group-M animals, and failed to reveal significant differences between the groups.

Water was available continually. Fresh fruits were provided 2–3 times a week, at random hours between 7am and 7pm, to avoid entrainment.

### Cognitive test

Throughout the entire experimental period, the availability of the default “free-food” access paradigm was contingent on the successful completion of the cognitive test. Whenever a monkey did not touch its monitor for over 30 min, a large gray square appeared in the center of the screen, indicating that a cognitive test would be initiated the next time the screen was touched. The delayed match-to-sample test (DMST) [46], with four standard pictures alternating on a random schedule, was used. Accordingly, a monkey was shown a “sample” picture, which it had to touch. After touching, the “sample” disappeared and a standard delay (20 sec) ensued after which the sample re-appeared with one of the other pictures (“choice”) and the monkey had to touch the “sample” shown earlier. If the “sample” was correctly chosen, the screen color turned green for 10 sec, while the standard 1 g chow pellet was dispensed. If the answer was incorrect, the screen turned red and stayed this way for 10 sec, with no reward provided. Immediately after, another sample picture was displayed, independent of whether the previous answer was correct or not. This sequence was repeated until a monkey acquired 15 correct choices, at which time the program automatically switched to the “free food” paradigm and the latter continued until a monkey would not touch the screen for at least 30 min. If, prior to completing 15 correct choices, the animal interrupted the DMST for over 5 min, the test was reinitiated, i.e., the earlier accumulated correct choices were “discarded”. This design allowed us to test cognitive performance around the clock, depending on the individual monkey-defined schedule and without inducing the stress of food deprivation. Documenting each touch of the screen, reaction time to touching a “sample” and “choice” pictures, correct or incorrect choice and test completion provided the cognitive test results for further analysis.

### Restricted timing of food access

During the experimental periods in CDL, the touch screen displayed the same moving target as in the “free-food” paradigm over the entire 24-h period. However, during a designated 12 hr period of restricted timing of food access, touching the on-screen target was documented but did not lead to the activation of the food dispenser. After the restricted food access period, a cognitive test icon was displayed and the animal had to complete the test in order to activate the “free-food” program, as described above, to begin a designated 12 h food access period.

The “premature” screen touches, i.e. those prior to the time when food became available, were documented and provided a measure of anticipatory activity of the animal. On the day when the scheduled time of food intake restriction was changed to an alternative 12 h period, the animal was allowed to consume food throughout the 24 h period, as per the regular combination of free-food and cognitive test paradigms, to avoid the stress of food deprivation. The 12-h periods of food restriction were scheduled according to individual's subjective day and subjective night, as detailed in the results section.

### Locomotor activity recordings and video monitoring

Each chamber was equipped with two infra-red light sources and two video cameras, each equipped with an infra-red pass filter. This allowed for uniform video-recording of the entire cage area, with picture quality independent of changes in environmental illumination (L vs. D vs. CDL). The continuous on-line actigraphic image analysis was conducted using custom-designed software [16], [20]. Changes in activity within each chamber were documented every 33 msec. A 15-sec integration period resulted in 240 data points per hour.

### Melatonin treatment

Melatonin was administered orally, inside dried fruit containing an individualized dose dissolved in 100 µL of ethanol. For each monkey, the overall experimental protocol included, as a first step, studying the dose-dependence of the effects of melatonin (2.5–20 µ/kg) on sleep in LD, as per our earlier studies. The results of these initial studies allowed us to confirm that low oral doses of melatonin [24], [25], starting with 5 µg/kg of body weight, were effective in all the animals described here. This was consistent with our earlier report on the sleep-promoting effects of low-dose melatonin in rhesus monkeys and two other diurnal macaque species [25], as well as in humans [26]. To test the entrainment to melatonin in CDL, it was administered daily (10 µg/kg), at the same clock time, starting with the chosen individual circadian time (CT10). Treatment typically continued until entrainment to a 24-h period of administration was documented, and for some time thereafter. The placebo preparation was identical, except for the lack of melatonin in the fruit. This small amount of fruit, even given at the same time of day, failed to entrain the animals.

### Suprachiasmatic Nuclei (SCN) Lesion

The SCN lesion was conducted in one group-M animal, M2, as part of a larger study on the effects of SCN lesions in rhesus monkeys. Here, we present the methodological approaches used, which allowed us to confirm complete ablation of the SCN in M2, directly relevant to the data presented here.

#### MRI Scanning for Surgical Planning

Prior to surgery, the subject was scanned in a 3T Philips MRI scanner, and a T1 weight magnetization-prepared rapid gradient echo (MP-RAGE) scan was obtained. To facilitate this, animal was anesthetized with ketamine hydrochloride (10 mg/kg initial; re-dosed at 5 mg/kg every 30 minutes for the procedure duration) and xylazine (0.125 mg/kg initial; re-dosed with 0.031 mg/kg at 30 minutes, if needed). The monkey was then placed in an MR-compatible stereotactic head holder fitted with ear bars containing vitamin E. This enabled the use of the ear bars as a landmark for surgical planning based on the MR images. Distances were calculated based on the following parameters in order to generate a series of stereotactic coordinates for the location of the suprachiasmatic nucleus. Anterior/Posterior Coordinate (AP): distance from the interaural line (MR image containing the center of the ear bars) to the target coronal slice containing the SCN. Medial/Lateral Coordinate (LT): distance from the midline between the cerebral hemispheres to a point directly above the SCN. To estimate the Superior/Inferior Coordinate (SI): distance from the pial surface and the surface of the corpus callosum overlying the SCN, down to the dorsal surface of the optic chiasm at midline, verified by the distance from the interaural line up to the optic chiasm. The coordinates to the anterior and posterior limits of the optic chiasm were also calculated. In M2, the coordinates were: AP = +24 mm, LT = 1.8 mm, and final SI was 0.5 mm dorsal to the *chiasm, as described below.*

#### Surgery

Under barbiturate anesthesia (0.25 mg/kg IV to effect), with the monkey in the same stereotactic machine used for the surgical planning scan, the *temporalis* muscle was mobilized and retracted to expose the skull. Using the surgical planning coordinates, a midline spanning bone flap was removed over the *optic chiasm* and reserved in sterile saline. With care taken to avoid the superior sagittal sinus and bridging veins, the *dura* was incised and retracted to expose the cortical midline. Midline coordinates and cortical surface coordinates over the SCN were then recorded and the midline of the hemisphere was gently retracted to visualize the surface of the *corpus callosum* and the anterior cerebral artery. Coordinates of the callosal surface were then taken and, if needed, the anterior cerebral artery gently mobilized and moved to avoid vascular damage. Then a bipolar concentric recording electrode was lowered on the midline toward the *optic chiasm*. Beginning when the electrode was about 5 mm above the *chiasm* a strobe light was flashed in the anesthetized subject's eyes to produce a visual evoked response. The electrode was advanced and this response mapped from the top to the bottom of the *chiasm* and then the same procedure was done moving in millimeter increments anterior and posterior to find the coordinates of the front and the back of the *chiasm*. Based on these coordinates the electrode was withdrawn and moved laterally to correspond to the location of the SCN and advanced until the first visual evoked potential was obtained on the surface of the *optic chiasm*. The electrode was then moved up until the lower of the two concentric tips was located 0.5 mm dorsal to the *chiasm* and an electrolytic lesion was made by passing between 1.0 and 1.5 mA of DC current for 60 seconds depending upon the case. The electrode was then moved to the other side of the midline and a mirror image SCN lesion was made. The *dura* was then closed, the bone flap sutured back into place and the muscle, fascia and skin closed in layers. Prophylactic analgesics were administered for 4 days and antibiotics for 7 days and the skin sutures removed between 7 to 10 days after surgery.

#### SCN lesion evaluation

At the conclusion of all testing, the animal was deeply anesthetized with sodium pentobarbital to a surgical level of anesthesia (0.25 mg/kg IV to effect). The monkey was then perfused through the ascending aorta with a 4% paraformaldehyde solution in 0.1 M phosphate buffer at pH 7.4 and 37°C. After removal, the brains were blocked, in situ, in the coronal stereotactic plane. One block containing the full extent of the hypothalamus was cryoprotected in phosphate buffer solution containing 10% glycerol and 2% DMSO for 2 days and transferred to 20% glycerol plus 2% DMSO for an additional 3 days. Brain was quickly frozen in −70°C 2-methyl-butane and then stored at −80°C. The tissue sections were cut in the coronal plane into 30 micron thick sections on a freezing sliding microtome. Sections were collected into interrupted series of every 10^th^ section so that the 30-micron sections in each series were spaced 300 microns apart. For anatomical evaluation of the SCN lesion one series was mounted and stained with thionin and the extent of the lesion was evalulated in comparison to the tissue sections from unoperated animals. In addition, other series were immunohistochemically stained to allow quantification of cells expressing two neuropeptides characteristic of different SCN compartments, arginine vasopressin (AVP, AbDSerotec, NC) and vasoactive intestinal peptide (VIP, a generous gift of Dr. Swaab, Netherlands Institute for Neuroscience). Detailed evaluation of the lesioned area from all three series in M2 suggested complete SCN ablation, without significant damage to the surrounding areas. The latter was based on the evaluation of the supraoptic (SON) and paraventricular (PVN) nuclei located next and caudal to the SCN. The observed lack of significant damage to the optic chiasm was consistent with the behavioral observations of preserved visual acuity during food acquisition (as described above) and ability to entrain to a light-dark cycle.

### Data analysis

#### Actigraphic data

Determination of the time of daily onset and offset of activity was based on visual inspection of double-plotted actograms, followed by detailed analysis of the areas of interest, with amplitude, density and duration of activity bouts being compared to typical daytime or nighttime behavior for individual animals. Our earlier extensive comparisons between polysomnographic and actigraphic sleep recordings conducted in the same circadian chambers described here allowed for the design of a software algorithm for actigraphic data analysis of sleep versus wakefulness in rhesus monkeys (Zhdanova et al, 2011). This allowed for reliable estimates of sleep duration and degree of sleep fragmentation (sleep and wake bouts number and duration) in the present study. Where needed, the corresponding video recordings were inspected for behavioral signs of wakefulness or sleep.

#### Circadian rhythm parameters

These included the length of intrinsic periods (τ), duration of daily activity, and four phase angles. The latter included: morning phase angle, Ψ_m_ (difference between lights on time and wake), evening phase angle, Ψ_e_ (difference between the lights off time and consolidated sleep period), lights-on-to-feeding phase angle, Ψ_lf_ (difference between the lights on time and the first feeding bout, defined as at least 5 consecutive pellets consumed, with inter-pellet interval less than 5 min), and wake-to-feeding phase angle (Ψ_wf_) (difference between wake-up time and the first feeding bout of that morning). Based on these daily measurements, mean (± SEM) intrinsic parameters were calculated for each animal, for each period of interest. The baseline chronotypes reported were assessed at the beginning of the longitudinal study, following an adaptation period of at least 2 months in the chambers (see Masuda & Zhdanova, 2010, for more details).

#### Food intake and Cognitive test parameters

*Measures of food intake and cogntive performance were evaluated* for the overall 24 h period, for an entire designated period or per hour of the designated period. Measures evaluated included: total food consumed, food consumed through “free-food” “cognitive test food” paradigms; success rate of cognitive performance (mean % correct choices in DSMT), mean reaction time to correct choice (RTc) and to incorrect choices (RTi), % tests completed (mean % of cognitive tests completed out of all tests initiated during the period). Designated periods included: light or dark period in LD, subjective day (activity period) or subjective night in CDL.

#### Spectral analysis

To characterize the spectral properties associated with the familial circadian disorder in group-M, the Fourier transform was used to calculate the relative power of all the frequencies of activity recording, present over selected periods of observation. To compare the results between the animals, the data were presented in two ways. First, as the power of all detected frequencies, with the sum of all the powers across considered as 100%. This relative power is reflected in 3D plots, although only 0–10 cycles per day are shown for visual clarity. Second, within the range of special interest, 0.5–5 cycles per day, i.e., corresponding to circadian and low ultradian periods, the data were normalized within this range, i.e. the sum of all the powers across this specific range for each individual animal was considered as 100% (Fig. 2 and 3).

To address the dynamic non-stationary nature of circadian and ultradian rhythms, we used the Morlet continuous wavelet transform (CWT), allowing for both time and frequency resolution of periodic processes that change over time [47]. Unlike in the windowed Fourier transform with fixed window size, the CWT window depends on the screened frequency, a distinct advantage when the duration of oscillations changes as a function of their frequency. The activity and food intake data series were analyzed using custom-designed wavelet analysis software [48]. The resulting wavelet ridges in the presented 3D graphs illustrate the relative strength (Z-axis) of a certain frequency (X-axis) at a certain time (Y-axis). The results are presented for locomotor activity data analysis (Fig. 6).

#### Statistical analysis

A linear mixed-model analysis, with the post-hoc Tukey multiple comparison test, was conducted to examine the effects of repeated treatments and changes in environmental conditions (Sigma Stat software, SPSS Inc. Chicago, IL). Linear regression was used to assess correlations, with R^2^ levels provided in the text. The data are presented as mean+/−SEM and the significance levels are provided in the text and figures. In radial graphs, differences between or within the groups are expressed as a relative percent change of the mean values compared, while the significance is indicated based on the raw data comparisons using the linear mixed-model analysis, as per the above. Unless otherwise indicated, the significance level in all the tests was set at p<0.05.
